# To pay or not to pay for artificial intelligence applications in radiology

**DOI:** 10.1038/s41746-023-00861-4

**Published:** 2023-06-23

**Authors:** Franziska Lobig, Dhinagar Subramanian, Michael Blankenburg, Ankur Sharma, Archana Variyar, Oisin Butler

**Affiliations:** 1 Bayer AG, Berlin, Germany; 2 Qlaar Limited, Oxford, United Kingdom

**Keywords:** Health policy, Medical imaging

## Abstract

Artificial Intelligence-supported digital applications (AI applications) are expected to transform radiology. However, providers need the motivation and incentives to adopt these technologies. For some radiology AI applications, the benefits of the application itself may sufficiently serve as the incentive. For others, payers may have to consider reimbursing the AI application separate from the cost of the underlying imaging studies. In such circumstances, it is important for payers to develop a clear set of criteria to decide which AI applications should be paid for separately. In this article, we propose a framework to help serve as a guide for payers aiming to establish such criteria and for technology vendors developing radiology AI applications. As a rule of thumb, we propose that radiology AI applications with a clinical utility must be reimbursed separately provided they have supporting evidence that the improved diagnostic performance leads to improved outcomes from a societal standpoint, or if such improved outcomes can reasonably be anticipated based on the clinical utility offered.

## Introduction

Artificial Intelligence-supported digital applications (AI applications) are expected to transform radiology. With more than 200 AI applications cleared by FDA, they have a broad range of use cases in radiology^1^. Image interpretation AI applications that help detect, quantify, and classify radiological abnormalities have been in the limelight. These applications aid in image processing and interpretation with greater accuracy and sensitivity by capturing subtle and complex patterns inconspicuous to the human eye or are burdensome to report by human readers^2^. By automating quantification and characterization of areas of interest, they help reduce variability between readers^3^. Their ability to discriminate lesions of interest with a greater degree of accuracy can aid with monitoring the progression of a disease^2^.

Although AI applications for image interpretation have caused the most excitement within radiology, non-interpretative AI applications also have the potential to play important roles in improving efficiency, safety, and quality. For example, deep learning using convolutional neural networks can successfully denoise 3D magnetic resonance images, which can improve scanning times without compromising on image quality^4^, to the advantage of improved protocoling, worklist prioritization, and reduced radiation exposure^3,5^.

Despite the promise AI applications hold in radiology, there are many barriers to the widespread adoption of AI. Integration of AI applications into radiology workflows can be complex and time-consuming. Radiologists and other users of AI software may resist to adopt to the new ways of working that some AI applications require. Hospitals and radiology practices may consider investment in radiology AI applications to be irrecoverable overheads that may not lead to direct cost-savings or additional revenue^6^.

The question on who will pay is critical for ensuring adoption of AI applications in radiology. Most mature healthcare systems use a prospective payment system for paying healthcare providers. Payments made under a prospective payment system for diagnostic radiology services are meant to cover the cost of conducting the imaging study and the associated reporting. Prospective payment systems are generally slow to account for increases in costs that are due to the adoption of innovations, which may not bear well for the adoption of AI in radiology^7^. On the other hand, payers may be concerned that paying separately for AI technologies may lead to over-use^8^. Therefore, healthcare systems need to find the right balance when determining which AI applications to pay for separately from the underlying imaging study, and which AI applications not to. Payers admit that this can be challenging^8^.

Although the payment pathways for radiology AI applications are in their nascent stages of development, a few radiology AI applications are already paid for separately in the Unites States (US). The National Health Service (NHS) in the United Kingdom (UK) and the National Health Insurance system of Japan also pay separately for radiology software applications that are offered as a Software-as-a-Service (SaaS)^9^.

In this article, we summarise the current reimbursement pathways for AI algorithms in radiology with specific focus on the US healthcare system, review the evidence basis for the radiology software applications currently reimbursed, and propose a conceptual framework to help determine when AI applications should be paid for separately from the underlying imaging study to incentivise adoption.

## Payment pathways already exist for radiology AI applications

The Centers for Medicare and Medicaid Services (CMS), the largest payer in the US, determines the coverage and payment for healthcare services furnished in inpatient and outpatient settings using the Inpatient Prospective Payment System (IPPS) and Hospital Outpatient Prospective Payment System (OPPS) respectively. Physician services provided in the office setting are paid through the Medicare Physician Fee Schedule (MPFS) (Table 1).

**Table 1:** Existing reimbursement pathways for AI applications in the US.

	Inpatient Prospective Payment System (IPPS)	Outpatient Prospective Payment System (HOPPS)	Medicare Physician Fee Schedule (MPFS)
Coding	International Classification of Disease (ICD)	Current Procedural Terminology (CPT) or Healthcare Common Procedure Coding System (HCPCS)	Current Procedure Terminology (CPT)
Payment mechanism for services	Medicare Severity Diagnosis Related Groups (DRGs)	Ambulatory Payment Classification (APCs)	Relative value units
Additional payment mechanism for AI applications	New Technology Add on Payment (NTAP)	New Technology APCs	New CPT codes
Criteria for qualifying for additional payment	Newness Cost Substantial clinical improvement	Newness Distinct procedure Medically reasonable and necessary	Medically reasonable and necessary

All three prospective payment systems require three components for successful reimbursement: coding, coverage, and payment. Coding refers to the existence of an alphanumerical code that describes the service or technology. Having an appropriate code is a pre-requisite for providers to bill payers for the service they provide. CMS must then make a positive decision to cover the service or technology, typically through a coverage policy, defining when a service or technology would be eligible for payment. The criteria for services or technologies to achieve coverage vary depending on the pathway, but CMS generally requires the service or technology to be ‘reasonable and necessary’. Finally, CMS must determine how much they would pay for the service or technology. Again, the method for determining payment varies across various Medicare pathways, but they typically consider the actual costs incurred by providers in providing the service, among other factors^10^.

The OPPS and MPFS pathways use Current Procedural Terminology (CPT) codes, a subset of Healthcare Common Procedure Coding System (HCPCS) codes to describe the service. CPT codes are created and managed by the American Medical Association (AMA). CMS can also create their own HCPCS codes for payment through OPPS. IPPS uses International Classification of Disease (ICD) codes.

Inpatient services are paid by grouping healthcare services that are similar from a clinical and resource-consumption standpoint into Medicare Severity Diagnosis Related Groups (MS-DRGs), each of which is assigned a payment. Novel healthcare services in the inpatient setting meeting certain criteria attract a temporary payment called the New Technology Add-on Payment (NTAP) in addition to the MS-DRG payment for a period of up to three years. NTAP was introduced in 2001 by the CMS to incentivise hospitals to adopt cost-increasing innovative technologies, since the payments for MS-DRGs lag actual costs by two to three years. NTAPs are temporary payments for a period of up to three years, the typical time for the cost of innovative technologies to reflect in the MS-DRG cost structure. Technologies approved under NTAP are paid additional amounts above the MS-DRG payment amount if they fulfill the following three criteria: the technology should be less than three years old; the cost of the technology should not be adequately covered under the MS-DRG; the technology should provide substantial clinical improvement over existing technologies. NTAP allows hospitals to bill CMS the lower of 65% of the amount by which the cost of the technology exceeds the DRG or 65% of the cost of the technology^11,12^.

OPPS uses a different grouping called Ambulatory Payment Classification (APC) to determine payments. New single-use physical medical devices can qualify for an additional payment for two to three years after FDA approval through transitional pass-through payments. CMS places new services that are unable to be placed in an existing APC in temporary APCs called New Technology APCs until sufficient claims data have been collected to allow CMS to assign the service to a routine clinical APC group that is appropriate in clinical and resource terms, which takes two to three years. To qualify under the New Technology APC, the service must satisfy the following criteria: the service must be truly new, it is not eligible for transitional pass-through payment, describe a distinct procedure, and is medically reasonable and necessary^8,12–15^.

The MPFS does not have a grouping; instead, payments are set for each CPT based on the Relative Value Units (RVUs), a measure of physician resource consumption for providing the service. AMA makes RVU recommendations to the CMS, which are then used by CMS to set the payments. New services require AMA to establish a new CPT code, to which CMS will allocate a payment based on the AMA RVU recommendation^11,12^.

Other countries have also started paying for radiology AI applications through existing prospective payment systems or special funding streams. In the UK, the MedTech Funding Mandate (MTFM) operates as a special policy mechanism to accelerate access to innovative medical devices, diagnostics, and digital products^16^. Technologies qualify if they have a positive NICE Medical Technology Guidance (MTG) or Diagnostic Guidance (DG), are cost-saving within three years of implementation and the total budget impact does not exceed £20 million in any of the first three years. MTFM does not provide direct funding for the technologies but mandates local payers to pay for the technologies^16^. In Germany, new technologies used in the inpatient setting can secure temporary add-on payments called New Examination and Treatment Methods (Neue Untersuchungs- und Behandlungsmethoden; NUB), whereas new technologies used in the ambulatory sector need to secure a listing in the Uniform Assessment Standard (Einheitlicher Bewertungsmassstab; EBM) catalogue after a positive health technology assessment by the Federal Joint Committee (Gemeinsamer Bundesausschuss; GBA)^17^. However, manufacturers cannot make a direct application, but must reply upon the hospitals and medical associations to make the applications in the inpatient- and outpatient settings respectively. In Japan, software applications approved by the Pharmaceutical and Medical Devices Agency (PMDA) are considered as medical devices and can be reimbursed through the medical device pathway using fee-for-service system^18^.

## A small proportion of applications are reimbursed

Over the last five years, eight of the 200-plus FDA-approved radiology software applications have been evaluated by CMS (Table 2). Two technologies have been rejected, while CMS has made positive coverage and payment decisions for the remaining six. One technology is covered through NTAP, five technologies through OPPS, and one through MPFS.

**Table 2:** Radiology software applications considered by CMS for coverage.

Pathway	Technology	Description	CMS decision
IPPS (NTAP)	Viz LVO (ContaCT)	Triage and notification software for stroke patients with suspected large vessel occlusion (LVO) in patients undergoing computed tomography (CT) angiography	NTAP status granted in 2020 and applicable until 2022
	Rapid ASPECTS	Software to calculate Alberta stroke programme early CT score (ASPECTS) from CT images in patients with stroke	Rejected in 2022
	Aidoc Briefcase for PE	Triage and notification software for pulmonary embolism (PE) in patients undergoing CT pulmonary angiography	Rejected in 2022
OPPS (APC)	HeartFlow	Software to quantify coronary flow from coronary CT images	New Technology APC granted in 2018; reassigned to clinical APC in 2022
	LiverMultiScan	Software to quantify liver pathology such as iron and fat content from liver magnetic resonance imaging (MRI)	New Technology APC granted from 2022
	Optellum Lung Cancer Prediction (LCP)	Software algorithm to produce a raw risk score for pulmonary nodules seen on chest CTs	New Technology APC granted from 2022
	MRCP+	Software to reconstruct biliary tree and quantify biliary obstruction from magnetic resonance cholangiopancreatography (MRCP)	New Technology APC granted from 2022
	Cleerly labs coronary analysis	Software application to determine the presence and extent of coronary atherosclerosis and stenosis from coronary CT angiography images	New Technology APC granted from 2023
MPFS	HeartFlow	Software to quantify coronary flow from coronary CT images	Included in the 2022 Medicare Physician Fee Schedule

Technologies used in the inpatient setting need to meet the substantial clinical improvement criterion, which can be challenging (Table 3). CMS has turned down two of the three NTAP requests made by vendors of triaging software applications used in the inpatient settings. ContaCT (also called Viz LVO), which detects large vessel occlusion (LVO) on computed tomography (CT) angiogram images, is the only inpatient-use radiology AI application to have successfully secured NTAP status^19,20^. Evidence presented for ContaCT showed that the application reduced time-to-diagnose large vessel occlusion. A historical comparison study additionally presented showed that the reduction in time-to-diagnosis also translated into superior clinical outcomes for patients, which was a crucial piece of evidence that helped convince CMS that ContaCT improves clinical outcomes.

**Table 3:** Clinical utility and evidence basis for the three radiology applications considered by CMS for NTAP.

Technology	Clinical utility	Trial design	Key clinical outcomes
ContaCT (Viz LVO)	Earlier identification of large vessel occlusion in patients with stroke	Retrospective study comparing ContaCT against standard reporting by neuro-radiologists	Average notification times were 7.32 minutes for ContaCT and 58.72 minutes for standard of care (mean difference 51.40 minutes; 95% confidence intervals 36.32–58.72 minutes)
		Pre-post analysis of a prospectively-maintained database from a large health system comparing patient outcomes before and after implementation of ContaCT	Post-ContaCT cohort had significantly better clinical outcomes and level of disability compared to the pre-ContaCT cohort, as measured by a lower 5-day NIH Stroke Scores (10.78 vs. 21.93; p = 0.02) and discharge modified Rankin Scores (2.92 vs. 4.62; p = 0.03)
Rapid ASPECTS	Automated calculation of Alberta stroke programme early CT score (ASPECTS) in patients with stroke	Three retrospective cohort studies and a concurrent-read crossover study comparing Rapid ASPECTS scoring and radiologist-reported ASPECTS score against expert consensus read	Rapid ASPECTS’ automated score had a higher level of agreement with pre-defined consensus than radiologists
		Prospective study done in Egypt comparing Rapid ASPECTS to standard care	Door-to-needle time for the standard care group was 36.8 minutes for Rapid ASPECTS compared to 52.3 minutes for standard care (p = 0.001)
Aidoc Briefcase for PE	Earlier identification of patients with pulmonary embolism	Retrospective study comparing the performance of Aidoc Briefcase with standard workflow	Mean time-to-notification with Aidoc Briefcase for PE was 3.9 minutes compared to 64.1 minutes for standard workflow (mean difference 60.2 minutes; 95% percent confidence intervals 32.7–87.6 minutes)
		Pre-post analysis from an unpublished retrospective study comparing patient outcomes before and after implementation of Aidoc Briefcase for PE	Lower mean length of stay for PE-diagnosed patients during the post-AI time period compared to pre-AI implementation (5.97 vs. 8.77 days; mean difference 2.80 days; p < 0.05)
		Pre-post analysis from an unpublished retrospective study comparing patient outcomes before and after implementation of Aidoc Briefcase for PE	30- and 120-day all-cause mortality were significantly reduced post-AI compared to the pre-AI implementation period (8.1% vs 7.7%, 15.5% vs. 9.6% respectively, p < 0.05)

However, in 2021, CMS turned down the NTAP request made for Rapid ASPECTS, a software to characterise brain tissue in patients with stroke (Table 3). RapidAI, the company behind Rapid ASPECTS, presented data from reader studies comparing Rapid ASPECTS scores with radiologist-reported ASPECTS score against an expert consensus read that served as the ground truth. The data showed that Rapid ASPECTS scoring had a higher level of agreement with consensus reads. RapidAI also presented additional data from a prospective study done in Egypt showing that Rapid ASPECTS reduces the time-to-treatment. However, CMS considered that high correlation between expert and Rapid ASPECTS scoring is not necessarily indicative of substantial clinical improvement. CMS also pointed out that there was no clinical evidence presented to support that the faster time-to-treatment shown for Rapid ASPECTS translates into superior clinical outcomes for patients^20,21^.

In 2021, CMS also turned down the NTAP request made for Aidoc Briefcase, a software for triage and notification of suspected pulmonary embolism (PE) cases in CT pulmonary angiography examinations (Table 3). Aidoc submitted data from a retrospective study showing that Aidoc Briefcase for PE reduced the time to notification. The vendor also presented pre-post analysis from an unpublished retrospective study comparing outcomes before and after implementation of Aidoc Briefcase for PE, that showed lower mean length of stay and mortality after implementation of the AI software. Even though the data supported the case for a substantial clinical improvement by showing not only improved time-to-diagnosis but also improved clinical outcomes, CMS considered that the studies did not compare Aidoc Briefcase with the existing standards of care, for example, prioritization through electronic health records. CMS concluded that the substantial clinical improvement criterion was not met.

The decisions made by CMS suggest that radiology AI applications in the inpatient setting intending to qualify for NTAP need to show that they not only offer clinical utility in terms of the ability to influence clinical care decisions, but also have clinical evidence showing that the improvement in clinical care decisions positively influence the clinical outcomes downstream. Undoubtedly, the quality of the studies also matters, as seen by the rejection of Aidoc Briefcase for PE for using a comparator that was not considered as the standard of care.

In contrast to the relatively high hurdle radiology AI applications have faced in the inpatient setting, AI applications have had relatively higher success in securing separate payment in the hospital outpatient setting since they did not have to meet the ‘substantial clinical improvement’ criterion.

The five applications covered through OPPS APCs have varied levels of evidence (Table 4). HeartFlow, a coronary flow quantification software, had robust evidence from a randomized controlled study and a non-randomized comparative study demonstrating that the application allows coronary flow to be quantified from CT coronary angiography images with comparable diagnostic performance and clinical outcomes as invasive pressure-wire based measurement, the existing standard of care. The studies also showed that HeartFlow lowers the cost of care since it helps avoid invasive interventions. HeartFlow is also recommended through the MedTech funding mandate by NHS England considering its cost-saving potential and is also paid separately through the medical fee schedule in Japan^16,22–27^. LiverMultiScan, a software that helps quantify liver pathologies from liver magnetic resonance imaging (MRI), had evidence from clinical studies showing that the technology detects liver diseases with a high degree of accuracy compared to gold-standard invasive liver biopsies, and that the parameters identified from LiverMultiScan analysis strongly predicted clinical outcomes in prospective studies^28–32^. Unlike HeartFlow, LiverMultiScan did not have direct real-world evidence that the application reduces the need for liver biopsies, but this was implicit since the application is proven to have a similar diagnostic information as liver biopsy without the risks associated with an invasive procedure. Optellum’s Lung Cancer Prediction (LCP) application showed greater accuracy in assessing and differentiating cancerous and benign pulmonary nodules compared to traditional risk detection models^33,34^ MRCP+, a software for use in magnetic resonance cholangiopancreatography (MRCP) to quantify biliary obstruction, had mainly data from a validation study using 3D-printed phantom models showing high accuracy in identifying biliary obstruction^35,36^. Finally, Cleerly labs coronary analysis software, used to quantify coronary obstruction from CT coronary angiography images without the need for invasive coronary angiography, had clinical evidence showing comparable diagnostic performance to quantitative coronary angiography, an invasive diagnostic test^37–40^.

**Table 4:** Clinical utility and evidence basis for the five radiology applications covered by CMS through OPPS.

Technology	Clinical utility	Trial design	Key clinical outcomes
HeartFlow	Quantification of coronary obstruction non-invasively and reducing the need for invasive coronary angiogram	PLATFORM, a prospective consecutive cohort study of HeartFlow compared to invasive coronary angiogram (ICA)	No difference in Major Adverse Cardiac Events at 1 year. In patients planned for ICA, mean costs lower for HeartFlow ($8,127 for HeartFlow vs. $12,145 usual care). ICA was avoided in 60%. QOL scores improved more in HeartFlow patients than in usual care patients
		FORECAST, RCT of 1,400 patients with stable chest pain comparing HeartFlow vs. routine care	There were 14% lower ICAs in the HeartFlow arm vs. routine care (p = 0.02). Total cost was lower (£1,605.50 in HeartFlow group vs. £1,491.46 in the routine care group)
LiverMultiScan	Quantification of liver parameters non-invasively and reducing the need for liver biopsy	Multiple prospective studies comparing LiverMultiScan with liver biopsy	Area under receiver operating curve ranged between 0.80 and 0.89 for diagnosis of non-alcoholic fatty liver disease, non-alcoholic steatohepatitis and cirrhosis
		Two prospective observational studies evaluating the prognostic value of LiverMultiScan parameters on the risk of liver associated clinical complications and death	LiverMultiScan parameters strongly predict clinical outcomes in patients with chronic liver disease
Optellum Lung Cancer Prediction (LCP)	Improved prediction of lung cancer risk	Prospective specimen collection with retrospective blinded evaluation study for validating the Optellum LCP model	Compared with traditional risk prediction models, Optellum LCP model was associated with improved accuracy (overall net reclassifications ranged from 0.30 to 0.58)
		Retrospective study to compare the risk prediction of Optellum LCP with standard risk prediction model	The area under the curve for Optellum LCP was 89.6% compared with 86.8% for the standard risk prediction model (p ≤ 0.005)
MRCP +	Improved diagnosis of biliary obstruction	Prospective study to determine accuracy, scan/rescan repeatability, and cross-scanner reproducibility of MRCP + , conducted in 40 subjects and two 3-D printed ‘phantom’ models	MRCP+ showed high accuracy (95% limits of agreement [LoA] (–1.1 to 1.0 mm), repeatability (LoA –0.4 to 0.4 mm), reproducibility across scanners (LoA –1.1 to 0.5 mm), and high inter- and intra-observer agreement based on the phantom models. MRCP+ detected biliary strictures and dilatations in the phantom with 76.6% and 85.9% sensitivity respectively and 100% specificity for both.
Cleerly labs coronary analysis	Improved coronary phenotyping	Retrospective studies comparing Cleerly labs quantitative coronary analysis with various current standards	Cleerly labs coronary analysis had high agreement (AUCs >0.80) with expert consensus read and quantitative coronary angiography, and higher diagnostic performance than myocardial perfusion imaging (AUCs 0.88–0.92 vs. 0.66–0.81)
		Retrospective study to evaluate interobserver variability in quantifying coronary plaque on coronary computed tomographic angiography, with Cleerly labs coronary analysis as reference standard	There was significant interobserver variability and high discordance with Cleerly labs coronary analysis when quantifying plaque composition

CMS did not evaluate the clinical evidence for these five technologies in detail since they did not have to meet the ‘substantial clinical improvement’ criterion unlike technologies in the inpatient setting. Despite the varied levels of evidence that these technologies have, they have something in common: most of them provide new diagnostic information which otherwise is impossible from the underlying imaging study or help improve the diagnostic performance of the underlying imaging study. For example, HeartFlow, LiverMultiScan and Cleerly labs coronary analysis software enable the radiologist or the referring physician to gain new diagnostic information that would not have been possible with the underlying standard imaging study, and by doing so, help avoid additional diagnostic procedures that are invasive. HeartFlow and Cleerly labs coronary analysis help quantify coronary obstruction, which otherwise require invasive coronary angiography, whereas LiverMultiScan enables quantification of liver pathology from MRI images without the need for liver biopsy. Optellum LCP provides superior diagnostic performance compared to standard risk prediction models, which has the potential to reduce unnecessary biopsies and improve detection of lung cancer, although there is no clinical evidence supporting this. Therefore, these applications offer implicit value to the patients and payers, which may have been a factor in CMS’s decision to cover these applications as separately payable service.

## A framework to evaluate whether to reimburse radiology AI algorithms separately

A question that is central to the adoption of AI in radiology is who should pay for AI. Payers have the unenviable task of balancing the need to pay for innovative cost-increasing technologies such as radiology AI applications, against the incremental budget impact of doing so.

It is well known that prospective payment systems do not effectively consider the additional costs associated with innovative technologies^41–45^. Prospective payment systems use historical cost data to determine payments for the service prospectively, without adequately considering the changes to the cost structure that innovative technologies may bring^46^. This frequently results in one of the following two scenarios: providers either bear the economic impact and therefore, lose money for each individual episode of care involving the innovative technology, or do not adopt the technology wary of the negative economic impact. Neither of these scenarios bode well for innovative technologies. There is empirical evidence that the introduction of a separate payment for innovative technologies that are expected to increase the cost of the relevant service positively influence adoption^47^.

Radiology AI applications are seeing an unprecedented pace of growth, with 210 applications approved as of November 2022, a steep increase from just six applications approved by end of 2017^48^. These applications vary significantly in terms of the incremental value they bring. Some applications help improve consistency in reporting whereas others offer new diagnostic information that human readers simply cannot deduce from studying the image. Applications also vary in terms of who benefits from the AI application. Some AI applications improve the efficiency of the reporting radiologist by automating measurements, and others offer a societal value by helping improve clinical outcomes or reducing the need for invasive and expensive imaging studies. Therefore, whilst adoption of radiology AI applications requires payers to consider paying for these AI applications separately, not all AI applications are equal, and therefore, not all AI applications may merit a payment separate from that of the underlying imaging study. Additionally, paying separately for radiology software applications has the potential danger of providers overusing technology for economic reasons. The 2002 decision by CMS to pay for computer-aided detection (CAD) in mammography led to an exponential growth in the use of CAD in mammography: while less than 5% of mammograms in the Medicare population were interpreted using CAD in 2001, the proportion ballooned to 74% by 2008^49^. The increased use of CAD had resulted in an incremental expenditure of $400 million per year. Subsequent real-world studies showed that CAD did not improve diagnostic performance of mammograms, which led CMS to remove the additional payment for CAD in mammography and bundle it into a single payment for mammography screening^49–51^. Therefore, there is a pressing need for payers to have clear criteria to determine which radiology AI applications must be separately payable, and which would be bundled within the underlying imaging study.

To our knowledge, payers have not developed such criteria specific for radiology applications yet. Instead, payers rely upon criteria of the underlying existing reimbursement pathways such as the NTAP in the US and MedTech funding mandate in England, which are meant for legacy technologies, and may not consider the nuances of cutting-edge technologies such as AI. Republic of Korea is the only advanced healthcare system to have considered developing such criteria, where the Health Insurance Review and Assessment (HIRA) service published a research paper in 2018 that discusses the circumstances when radiology AI applications must be reimbursed separately. The paper suggests that radiology AI applications that provide new diagnostic information such as the ability to predict prognosis or serve as new imaging biomarkers may merit separate reimbursement provided they have evidence on clinical effectiveness or cost-effectiveness. The paper is non-binding and has not turned into firm policy action yet^52^.

In developing criteria to determine which radiology AI applications to pay for, we believe there are two aspects that payers must consider. The first is the nature of benefit that the applications bring. As discussed before, radiology AI applications are heterogeneous in their nature and offer a broad range of benefits. They also vary in who they benefit. Therefore, the nature of the benefits as well as the stakeholders who benefit from the applications must be considered when determining who will pay for the application. The second key aspect is the availability of evidence to support the existence of the benefit. It is reasonable for payers to expect evidence that radiology applications aiming to improve diagnostic performance of an imaging study, for example, also bring direct or indirect evidence that the improved diagnostic performance will lead to an improvement in clinical outcomes with sufficient certainty.

**Fig. 1:**
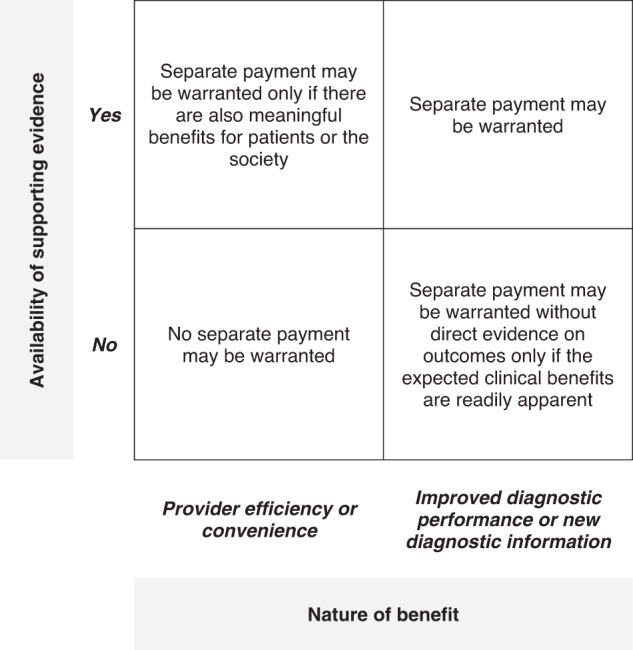
Proposed framework to determine whether to pay for radiology AI applications separately or additionally to underlying imaging studies.

Based on these two aspects, we propose a framework to determine the reimbursability for radiology AI applications that payers as well as technology developers might find useful (Fig. 1). The first dimension, represented in the X-axis in the framework, classifies the benefit the radiology application brings, into whether it improves provider efficiency or convenience, or whether it improves the diagnostic performance or provides new diagnostic information that is otherwise not possible from the relevant imaging study. The second dimension, represented in the Y-axis, considers whether the AI application has evidence supporting the improvement of clinical and/or economic outcomes.

Applications such as automatic measurement algorithms that solely improve the efficiency or convenience of the radiologist or the healthcare provider (represented in the two left quadrants in Fig. 1), generally may not require or merit additional payment since the nature of the benefit itself is self-evidence to providers and is a sufficiently robust incentive for the providers to use the application. Even if there is supportive evidence for these applications, the evidence would likely be of an economic nature, and not show clinical benefits. Such economic benefit would also accrue to providers, and therefore, such applications may not warrant separate payment irrespective of the evidence basis. However, there may be some exceptions to this. For example, an AI application that checks images for abnormalities as they are acquired may help improve provider efficiency, but in addition, may also have a large impact on patients by ensuring the right imaging protocol is used, and thereby, reducing the need for repeat imaging studies. Similarly, in countries such as the United Kingdom, where healthcare capacity can be a constraint, an AI application that reduces scan time or reporting time may help not just the provider, but also have broader benefits to patients and the society by reducing waiting times for imaging studies. In such circumstances, payers may decide to incentivise the adoption of AI applications improving efficiency by paying for them separately.

Conversely, applications that improve diagnostic performance of the underlying imaging study or provide new diagnostic information that is otherwise impossible to obtain with the underlying imaging study merit consideration for additional payment (represented in the two right quadrants in Fig. 1). Optellum’s Lung Cancer Prediction application, for example, which improves the diagnostic performance of pulmonary CT studies by providing a more accurate risk-prediction score, and HeartFlow software, which calculates coronary flow parameters from CT coronary angiography studies that otherwise require invasive coronary angiography, may fit into these quadrants. Providers require an economic incentive to adopt such AI applications as they increase the total cost of care, and since the benefits, in the form of potential improvement in patient outcomes or reduction in downstream costs, do not accrue to the providers, but to the broader society. In fact, radiology AI applications that reduce the need for additional investigations may reduce provider revenues. It is apparent that payers must be obliged to pay for these applications, especially if they also have evidence supporting the existence of clinical and/or economic benefit (represented in the top right quadrant in Fig. 1).

Whilst no doubt manufacturers must aim to develop such evidence, we need to recognise that developing such evidence for diagnostics may be challenging sometimes. Radiology AI applications do not have the strong intellectual property benefits that pharmaceuticals enjoy. Additionally, developing long-term evidence on clinical outcomes may be challenging for some diagnostic applications if this requires patients to be followed through diagnosis, treatment, and subsequent clinical outcomes^53^. In such circumstances, where applications offer a clear clinical utility in terms of improvement in the clinical decision-making, but there are real challenges developing direct evidence that the improvement in clinical decision-making results in improved clinical outcomes, payers may have to consider paying for the applications despite the limitations in evidence. For example, it would be unreasonable to expect each breast cancer detection application to come to the market with direct evidence that the improved detection also improves survival, since this evidence would take years to develop. In this case, denying reimbursement because of the lack of direct evidence on clinical outcomes would not only delay or deny access to a transformational technology, but also be unnecessary since it may be readily apparent from literature that detecting cancer at a sooner stage or cancer at a smaller size is directly correlated with improved survival rates and health outcomes. Similarly, the benefits of an AI application that helps characterise liver tissue from magnetic resonance images with a comparable degree of diagnostic performance against the gold-standard liver biopsy are readily apparent without having to conduct a clinical study to prove that the AI application helps reduce healthcare costs or avoid adverse events associated with biopsy. Hence, we argue that for applications that improve diagnostic performance or those that provide new transformational diagnostic information, payers must consider separate payment even without supporting clinical evidence if the anticipated clinical and/or economic benefits are meaningful and are readily apparent (represented in the bottom right quadrant in Fig. 1).

We recognise that the framework proposed is conceptual, and payers would need to consider several important issues before determining their payment policies for radiology AI applications. Payers would have to define what constitutes a meaningful benefit – as opposed to just a statistically significant improvement – and what the minimum level of evidence is to support the existence of such a benefit. Valuing healthcare interventions can be complex, and radiology AI applications are no different. Cost-offsets may be easy to quantify, but the value of earlier or more accurate diagnosis may be much harder to value. Furthermore, improving diagnosis may increase total healthcare costs by increasing downstream spend on additional investigations or treatments, and therefore, payers may have to consider not just the value, but also potential budget impact. The value of an AI application may differ substantially between healthcare systems, and therefore, payers will have to evaluate radiology AI applications within the context of their own healthcare systems and determine whether and how to pay for these applications. For example, in a healthcare system with poor access to specialist radiologists, a radiology AI application may help improve diagnostic accuracy and clinical outcomes, whereas in a healthcare system with good access to specialist radiologists the same application may help improve efficiency of the reporting radiologist more than diagnostic accuracy. Payers also need to consider several ethical aspects when determining their payment policies for AI applications such as the perspective they must take when evaluating applications, willingness-to-pay thresholds and whether to consider paying more for applications aimed at diagnosing rare conditions.

Most of these issues we have highlighted above are not necessarily unique for radiology AI applications: these issues have applied historically to other healthcare technologies such as pharmaceuticals. However, payers must also recognise that the frameworks and evidence standards applicable for pharmaceuticals cannot be directly transposed for radiology AI applications, since there are multiple differences between them. AI applications are iterative in nature and evolve over time. Therefore, a single large trial may become irrelevant by the time the results are available. Randomised controlled trials may be the gold-standard for interventions such as pharmaceuticals, but for diagnostics, payers must recognise that high quality real-world studies play a key role in generating robust evidence that is otherwise difficult or impossible to study^54^.

Reimbursement coverage policies are critical determinants of adoption, and therefore, have a large impact on product development decisions^55^. It is paramount for payers to develop clear criteria for determining coverage of radiology AI applications and apply this consistently when making decisions. Additionally, payers and technology developers must engage in early discussions on evidence requirements based on target product profiles to ensure development of the right evidence. In healthcare systems where existing processes allow only healthcare providers or medical associations to initiate reimbursement requests, payers must consider modifying these processes to allow technology manufacturers to make direct reimbursement requests.

## Conclusion

Imaging AI applications are expected to transform radiology. However, widespread adoption of these applications requires providers to have the motivation and incentives to adopt. For some radiology AI applications, the benefits of the application such as improved efficiency or lower costs themselves may sufficiently serve as the incentive for providers to adopt. For others, especially those that increase costs or reduce revenue for the provider, the economics may serve as barriers to adoption, and therefore, payers using prospective payment systems must consider reimbursing the AI technology separate from the cost of the underlying imaging studies to optimise health outcomes and societal value.

In such circumstances, it is important for payers to develop a clear set of criteria to decide which AI applications should be paid for separately. The framework we have proposed may help serve as a guide for payers aiming to establish such criteria as well as technology vendors developing radiology AI applications. As a rule of thumb, we propose that radiology AI applications that improve diagnostic performance of the imaging study or provide new diagnostic information that did not hitherto exist, must be reimbursed separately provided they have evidence supporting that this improved diagnostic performance leads to improved outcomes from a societal standpoint. Additionally, we argue that payers must consider separate payment for radiology AI applications that improve diagnostic performance substantially or provide transformational new diagnostic information even without supporting clinical evidence if the anticipated clinical and/or economic benefits are large, and reasonably certain.

Considering the unique nature of radiology AI applications, payers must also consider defining evidence standards that are acceptable, recognising that high quality non-randomized studies may offer robust real-world evidence where randomized controlled trials are difficult or impossible to conduct. Payers must consider streamlining processes to allow technology manufacturers to make direct reimbursement requests. Finally, since reimbursement can be a critical determinant of adoption, payers and technology developers must engage in early and direct discussions on evidence requirements based on target product profiles to ensure development of the right evidence.

### Reporting summary

Further information on research design is available in the Nature Research Reporting Summary linked to this article.

## Supplementary information

Reporting Summary

## Data Availability

No datasets were used in the development of this manuscript.
